# Impact of Soil Salinity on the Structure of the Bacterial Endophytic Community Identified from the Roots of Caliph Medic (*Medicago truncatula*)

**DOI:** 10.1371/journal.pone.0159007

**Published:** 2016-07-08

**Authors:** Mahmoud W. Yaish, Abbas Al-Lawati, Gerry Aplang Jana, Himanshu Vishwas Patankar, Bernard R. Glick

**Affiliations:** 1 Department of Biology, College of Science, Sultan Qaboos University, Muscat, Oman; 2 Department of Biology, University of Waterloo, Waterloo, Ontario, Canada N2L 3G1; Free University of Bozen/Bolzano, ITALY

## Abstract

In addition to being a forage crop, Caliph medic (*Medicago truncatula*) is also a model legume plant and is used for research focusing on the molecular characterization of the interaction between rhizobia and plants. However, the endophytic microbiome in this plant is poorly defined. Endophytic bacteria play a role in supplying plants with the basic requirements necessary for growth and development. Moreover, these bacteria also play a role in the mechanism of salinity stress adaptation in plants. As a prelude to the isolation and utilization of these bacteria in Caliph medic farming, 41 bacterial OTUs were identified in this project from within the interior of the roots of this plant by pyrosequencing of the small ribosomal subunit gene (16S rDNA) using a cultivation-independent approach. In addition, the differential abundance of these bacteria was studied following exposure of the plants to salinity stress. About 29,064 high-quality reads were obtained from the sequencing of six libraries prepared from control and salinity-treated tissues. Statistical analysis revealed that the abundance of ~70% of the OTUs was significantly (*p* ≤ 0.05) altered in roots that were exposed to salinity stress. Sequence analysis showed a similarity between some of the identified species and other, known, growth-promoting bacteria, marine and salt-stressed soil-borne bacteria, and nitrogen-fixing bacterial isolates. Determination of the amendments to the bacterial community due to salinity stress in Caliph medic provides a crucial step toward developing an understanding of the association of these endophytes, under salt stress conditions, in this model plant. To provide direct evidence regarding their growth promoting activity, a group of endophytic bacteria were isolated from inside of plant roots using a cultivation-dependent approach. Several of these isolates were able to produce ACC-deaminase, ammonia and IAA; and to solubilize Zn^+2^ and PO_4_^-3^. This data is consistent with the predicted occurrence (based on cultivation-independent techniques) of these bacteria and provides some insight into the importance of the endophytic bacteria in Caliph medic when grown under normal and saline conditions.

## Introduction

Endophytic bacteria refers to those species that are able to grow within plant tissues without showing disease symptoms, and survive by forming a symbiotic relationship with the host plant [1]. Endophytes can promote plant growth by increasing the availability of some nutrients, such as nitrogen, phosphorus, iron and zinc; by synthesizing growth hormones, such as indole-3 acetic acid, cytokinins and gibberellic acids [2]; and by producing 1-aminocyclopropane-1-carboxylic acid (ACC) deaminase, an enzyme responsible for the cleavage of ACC, which is the immediate precursor of the hormone ethylene in all higher plants [3,4]. Endophytic bacteria are important for both the routine growth and the developmental processes of plants, as well as when plants experience biotic and abiotic stresses including salinity [5,6]. Under saline conditions, some endophytic microorganisms ameliorate the stress in plants by synthesizing osmoprotectant molecules, such as proline and/or trehalose [7], quaternary ammonium compounds in the cytoplasm [8,9], volatile organic molecules [10], and exopolysaccharides [9,11].

Salinity can severely affect plant health and yield by causing an imbalance in nutrient uptake, by increasing the negative osmotic water pressure on plant cells [12,13], and by reducing the availability of nutrients in the soil [14]. Recent research, however, has revealed that plant growth-promoting bacteria including both endophytic and rhizospheric bacteria can improve the survival chances and performance of plants under saline conditions [6,15,16]. For example, the inoculation of cucumber [17] and canola [3] with *Pseudomonas putida* UW4 has been found to enhance plant growth under saline conditions. Likewise, the treatment of pepper seedlings with *Brevibacterium iodinum*, *Bacillus licheniformis* and *Zhihengliuela alba* halotolerant bacteria reduced salt-induced ethylene synthesis and, as a result, promoted plant performance under the same conditions [18].

Alfalfa (*Medicago sativa*) and Caliph medic (*Medicago truncatula*) are important fodder crops worldwide, but their production has been severely reduced in recent decades due to soil salinity [19,20,21]. In addition, *M*. *truncatula* is considered to be a model legume plant for molecular research on rhizobium-legume symbiosis [22]. For this reason, *M*. *truncatula* was chosen for the current study into the impact of soil salinity on endophytic community richness in Caliph medic and other related species such as alfalfa.

Although numerous bacterial taxa were previously identified as endophytes in *M*. *sativa*, endophytes have not been studied in *M*. *truncatula*. For example, in addition to the well-known *S*. *meliloti*, a detailed study using Terminal-Restriction Fragment Length Polymorphism (T-RFLP) analysis, quantitative PCR and sequencing of the 16S rRNA gene showed that the root system of alfalfa was enriched with a wide range of species classified under the *Sphingomonadaceae* and *Methylobacteriaceae* bacteria families [23]. Other studies have shown that the bacteria community also includes *Bacillus megaterium* [24], *Brevibacillus choshinensis* and *Microbacterium trichothecenolyticum* [25], *Endobacter medicaginis* [26] and *Micromonospora* sp. [27].

Describing a microbial community structure, in which each bacterial member of the community is isolated, is not currently possible because we lack the knowledge to cultivate a large percentage of these microbes. In addition, determining the change to the community structure when the community is exposed to salinity stress requires a differential quantitative method in order to individually estimate the taxon abundance within the community. Currently, the use of the *16S rRNA* gene sequencing in studying the microbial community structure is much more comprehensive than using culture-based approaches [28,29]. Therefore, in the project reported herein, a next-generation pyrosequencing method was used to characterize the structure of the endophytic community and to estimate the corresponding changes that occur in response to salinity stress in *M*. *truncatula* root tissues. These changes may provide an insight into the role of the endophytic microbial community in salinity tolerance in Caliph medic.

## Material and Methods

### Soil analysis

The physical and chemicals properties of the soil, including the electrical conductivity (E.C.) and the pH, were measured as described previously [30] by the Ministry of Agriculture and Fisheries’ soil analysis laboratories in Jomah, Oman.

### Plant materials

Caliph medic (*Medicago truncatula*) seeds were surface-sterilized with 75% ethanol for 10 minutes and then with a 5% sodium hypochlorite solution for five minutes. Next, caliph medic seeds were rinsed three times with sterile distilled water. Seeds were planted in pots containing soil collected from fields used to grow different *Medicago* species such as alfalfa and caliph medic, which were located at the coordinates 23°39'42.5"N and 58°00'34.0"E, Jomah, Oman. Plants were grown in two–liter pots and placed in the field, where the day and night temperatures were respectively 28±2°C and 20±2°C, and in the natural daylight. Six pots were used, with each pot containing at least 10 seedlings. Three of the pots were used for the control treatment, while the other three pots were used for the NaCl treatment. The plants used in the control treatment were watered weekly with distilled water, while the salinity-treated plants were watered for the first two weeks with distilled water and then with increasing levels of saline solution on a weekly basis, starting with 50 mM, followed by 75 mM and then 100 mM NaCl for two successive weeks.

Plant roots were collected from the seedlings 50 days after planting. Roots of 10 different seedlings were pooled and considered as one replicate. Three replicates of the control and the NaCl treated pools were used in this experiment. The collected roots were surface-disinfected in line as described previously [31]. Briefly, a pool of roots from the control and the salinity-treated plants were separately washed in running water, then disinfected by treatment with 5.25% bleach for 3 minutes followed by 3% hydrogen peroxide solution for 3 minutes, and then washed twice with sterile distilled water containing a 10% solution of Tween 20. Finally, the roots were rinsed twice with sterile distilled water. To examine the surface disinfection efficiency, a sample of the roots from each pool was planted in solid TSA medium for one week at 28°C. Subsequently, the plates were examined for the presence of microbial-growing colonies. The surface-disinfected roots were flash-frozen in liquid nitrogen and kept at -80°C in a freezer until they were used for DNA extraction. The roots were grounded in liquid nitrogen using a sterile mortar and pestle. The DNeasy Plant Maxi Kit (Qiagen) was used to extract the total DNA, which contained both the plant cellular DNA and the microbial DNA of the endophytic community.

The bacteria communities were fingerprinted according to the ribosomal DNA (16S rRNA) sequences, using the pyrosequencing method (GS FLX+) and a 454 platform sequencer (Roche). The V3-V4 16S rRNA was amplified by PCR-fusion [32] using universal oligonucleotides (S1 Table). The PCR products were purified using AMPure9 beads and quantified using a Picogreen assay [33], while the CD-HIT-OTU (version 454–0.0.2) was used to assemble the raw data *de novo*. The amplicons were sequenced and assembled using the next-generation sequencing facilities at Macrogen, Inc. (Seoul, the Republic of Korea).

### Isolation, identification and characterization of endophytic root bacteria

The endophytic bacteria were isolated from the surface sterilized roots of the Caliph medic plants grown under normal and saline conditions as previously described [6,31]. The bacterial strains were identified based on the 16S rDNA gene sequences. The 16S rDNA genes were amplified from the genomic DNA by PCR using the 27F and 1492R primers [34]. ACC deaminase activity in the newly isolated strains was determined using the procedure of Penrose and Glick [35]. The ability to produce IAA and similar compounds using the L-tryptophan was determined as previously described [6,31]. The capacity of the strains to produce ammonia was measured as previously described [36], while the ability to solubilize PO_4_^3-^ and Zn^2+^ using the Ca_3_(PO_4_)^2^ and the ZnO insoluble salts in the Pikovskaya’s agar media respectively, was determined using previously described methods [37].

### 16S rRNA sequence analysis

Raw data were demultiplexed using barcode sequences without allowing for any mismatch (Macrogen’s in-house software). Short reads were filtered, while tails and the reads that were too long were trimmed. Duplicates and chimeric reads were removed, with the resultant reads clustered with 100% identity using CD-HIT-DUP software [38]. Using a greedy algorithm [39], the remaining representative high-quality reads from the non-chimeric clusters were clustered into Operational Taxonomic Units (OTUs) with a similar cut-off identity at the species level as follows: for species 98%, for genus 94%, for family 90%, for order 85%, for class 80% and for phylum 75%.

Raw data were classified based on the barcode sequences of each sample. In order to find the best match, each sequence was compared (locally and globally) to the sequences available in the SILVA database. QIIME 1.8.0 software [40] was used to produce the OTU count. The similarity between the read sequences was examined in order to identify the OTUs as well as carry out statistical analysis on the diversity and evenness of the sample species. The Shannon, Simpson and Chao 1 indices were used to study the biodiversity based on the richness of the species and to estimate the abundance-based richness within the community [41].

The raw abundance value was used without rarefying for analyzing diversity statistics community richness and diversity using the biom files and the QIIME software. The goods coverage was also calculated using the calculator provided by software QIIME.

Each group (salinity and control treatments) was composed of three biological replicates and the validation of the data was based on *p* ≤ 0.05. With regard to the QIIME software, the Mann-Whitney U test, as a bootstrap version equal to 2,000 times, was used. The *p*-value was corrected by the Bonferroni procedure for multiple comparisons [42,43]. The complete linkage between the hierarchical cluster analysis and the visualization of the abundance values as a heat map were carried out using the PermutMatrix software [44]. The hierarchical cluster was set at a complete linkage and the dissimilarity was calculated based on the Euclidean distance. A neighbor-joining phylogenetic tree was built using the Mega software package 5.0 [45] and the default settings.

Principal Coordinate Analysis (PCoA) [46] analysis was used as an ordination-based approach to illustrate the variation between the bacterial community compositions in response to salinity treatment using the Past 3 software package [47] and the Gower’ distance matrix [48]. In addition, analysis of similarity test (ANOSIM) [49] and the Gower distance matrix index were used to assess the pairwise comparisons of significant differences between the microbial communities. The *p*-value was recalculated based on the Bonferroni significance.

The 16S rDNA sequences were deposited in GenBank/EMBL/DDBJ under the accession numbers KU587127-KU587167 and KX395941-KX396022.

## Results and Discussion

### Salinity stress impaired the growth of seedlings

**Fig 1:**
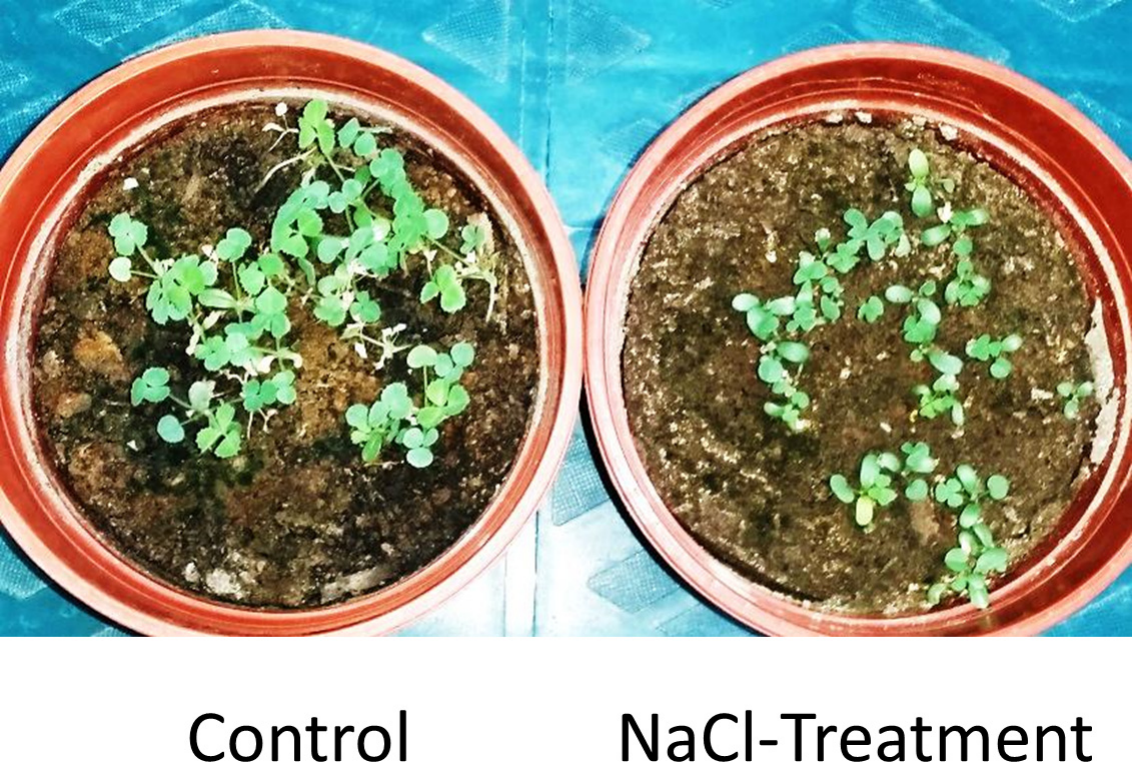
The influence of salinity treatment on Caliph medic seedlings.

Plant species vary in their ability to cope with salinity stresses. Although Caliph medic is considered to be a moderately salinity-tolerant plant [50], this tolerance depends on the growth stage and the genotype of the plant [20,51]. In this study, we have identified endophytic bacteria from Caliph medic at the seedling stage; therefore, the total endophytic microbial community of this plant may not be limited to those microbes described in this study. In comparison with the control experiment, the effect of the soil salinity stress was evident in the performance of the seedlings (Fig 1).

This is quite normal since the soil salinity level measured as electrical conductivity (E.C.) dramatically increased due to the treatment from 1.23 (S.D. ±0.22) to 14.36 (S.D. ±0.4). That increase in the E.C. not only significantly increased the Na^+^ and Cl^+^ (*p* ≤ 0.05) (Table 1), but it also increased the soluble sulphate in the soil, presumably because sodium interacts with sulphate to form a soluble salt [52].

**Table 1:** Physicochemical properties of the soil used to grow Caliph medic seeds.

Soil physicochemical properties	Average Content		*p*- value
	Control	NaCl-treatment	
EC (dS/m)	1.23	14.40	0.001
pH	7.30	7.30	1.000
Na (meq/l)	11.16	81.27	0.001
K (meq/l)	2.86	3.57	0.130
Ca (meq/l)	18.40	19.51	0.030
Cl (meq/l)	15.03	116.33	0.001
HCO_3_ (meq/l)	7.24	1.17	0.001
SO_4_ (meq/l)	8.15	11.78	0.001
Gravel %	18.17	18.14	0.970
Sand %	93.04	94.60	0.100
Silt %	3.20	3.20	1.000
Clay %	2.30	2.30	1.000
CaCO_3_	22.10	21.89	0.001
N %	0.06	0.06	1.000
P (ppm)	89.70	110.80	1.000
K (ppm)	330.00	390.00	1.000

Soil analysis revealed a significant (*p* ≤ 0.05) reduction in the bicarbonate and a slight reduction in the calcium carbonate content of the soil in response to salinity treatment (Table 1). This is consistent with the observation that soil salinity reduces the global soil carbon stocks due to a reduction in microbial activity and, hence, in organic carbon decomposition rates [53].

### 16S rRNA sequencing uncovered the presence of a divergent endophytic bacterial community

Plants interact with the surrounding environment, including soil inhabitants such as bacteria; however, these microorganisms have only a minor effect on the structure of the endophytic bacterial community [54]. Since it was not possible to exclusively extract the microbial genomes from the root tissues, the total DNA, including the plant genomic DNA, was extracted from these tissues and used for bacterial DNA barcoding. Pyrosequencing of the resultant 16S rRNA libraries yielded a total of 119,381,266 pb from three control and three treatment libraries. The nucleotide sequences obtained from the control and the treated libraries were assembled into 167,698 reads, 94% were coded for high quality barcode sequences with an average length of ~712 bp. After the removal of the mitochondrion and chloroplast related sequences from the OTU list, a total of 29,064 high quality 16S rRNA sequences were obtained from the six libraries including 2,274 and 26,790 belonged to the control and the NaCl-treated libraries, respectively. The relatively high number of 16S rRNA reads obtained from the communities isolated from the roots grown in saline conditions is due to the presence of some overrepresented OTUs in these communities. For example, the reads associated with the three bacterial species *Thalassospira povalilytica*, *Castellaniella hirudinis* and *Pseudomonas stutzeri* account for 39.3% (10,528) of the total reads obtained from the sequencing of these libraries (Table 2).

**Table 2:** Bacterial OTUs identified from Caliph medic roots based on 16S rRNA DNA sequences and the mean of abundance in the libraries prepared from the control and salinity-treated plants. Significant enrichment (*p* ≤ 0.05) of a certain OTU was calculated based on three biological replicates. The OTUs were arranged based on the descending *p*-value.

OTU	Mean of abundance		*p*-value	OTU	Mean of abundance		*p*-value
	Control	Treatment			Control	Treatment	
*Brucella inopinata*	70.7	0	0.036	*Pseudoxanthomonas mexicana*	4.7	0.4	0.053
*Sphingobium xenophagum*	56.7	3.0	0.037	*Acidovorax soli*	4.0	0	0.055
*Tistrella mobilis*	12.0	0.0	0.041	*Enterobacter* sp.	0.4	744.4	0.055
*Streptomyces variabilis*	0.0	6.7	0.041	*Cellvibrio diazotrophicus*	3.4	85.7	0.057
*Enterobacter kobei*	0.0	79.4	0.043	*Pseudomonas stutzeri*	6.0	841.0	0.058
*Thalassospira xianhensis*	0.0	19.7	0.043	*Enterobacter cloacae*	5.0	704.0	0.058
*Pseudoxanthomonas broegbernensis*	6.4	0.0	0.044	*Achromobacter pulmonis*	0.7	10.7	0.058
*Pseudomonas resinovorans*	3.4	8.7	0.045	*Pseudomonas borbori*	3.4	474.0	0.058
*Marinobacter gudaonensis*	0.0	31.0	0.045	*Rhizobium rosettiformans*	40.4	244.4	0.063
*Shinella granuli*	85.0	4.7	0.045	*Pseudomonas indica*	3.0	610.4	0.067
*Fluviicola* sp.	0.0	13.0	0.045	*Salinicola salarius*	2.7	46.7	0.067
*Sphingopyxis macrogoltabida*	7.0	1.4	0.047	*Rheinheimera aquimaris *	62.7	765.0	0.068
*Inquilinus* sp.	0.0	67.0	0.048	*Pseudomonas aeruginosa*	185	330.7	0.068
*Halomonas lutea*	2.0	118.7	0.049	*Methylophaga* sp.	1.4	16.7	0.071
*Flavobacterium glycines*	0.0	64.4	0.049	*Halomonas* sp.	92.4	628.7	0.072
*Pseudomonas pseudoalcaligenes*	0.0	4.4	0.050	*Pseudomonas mendocina*	1.4	260.4	0.073
*Thalassospira povalilytica*	2.4	1351.7	0.051	*Sphingobacterium thalpophilum*	5.7	0.0	0.185
*Beijerinckia fluminensis*	46.7	0.0	0.052	*Rhizobium halotolerans*	0.0	5.7	0.190
*Marinobacter nanhaiticus*	17.0	62.0	0.053	*Sphingomonas koreensis*	3.7	2.4	0.583
*Pseudomonas* sp.	21.4	5.4	0.053	*Methylobacillus flagellatus*	2.4	2.0	0.893
*Castellaniella hirudinis*	0.0	1316.7	0.053				

The taxonomy abundance ratio and the sequence similarity analysis of the 16 rRNA using BLAST showed that about 99% of the 16S rRNAs were classified under the *Proteobacteria* phylum, while the rest were classified under the *Actinobacteria* and *Bacteroidetes* phyla. The *Actinobacteria* phylum included the *Streptomyces* family; the *Bacteroidetes* phylum included the *Flavobacteria and Sphingobacteriia* families; and the *Proteobacteria* included 14 different families including *Chromatiaceae*, *Enterobacteriaceae*, *Pseudomonadaceae* and *Rhizobiaceae*.

**Fig 2:**
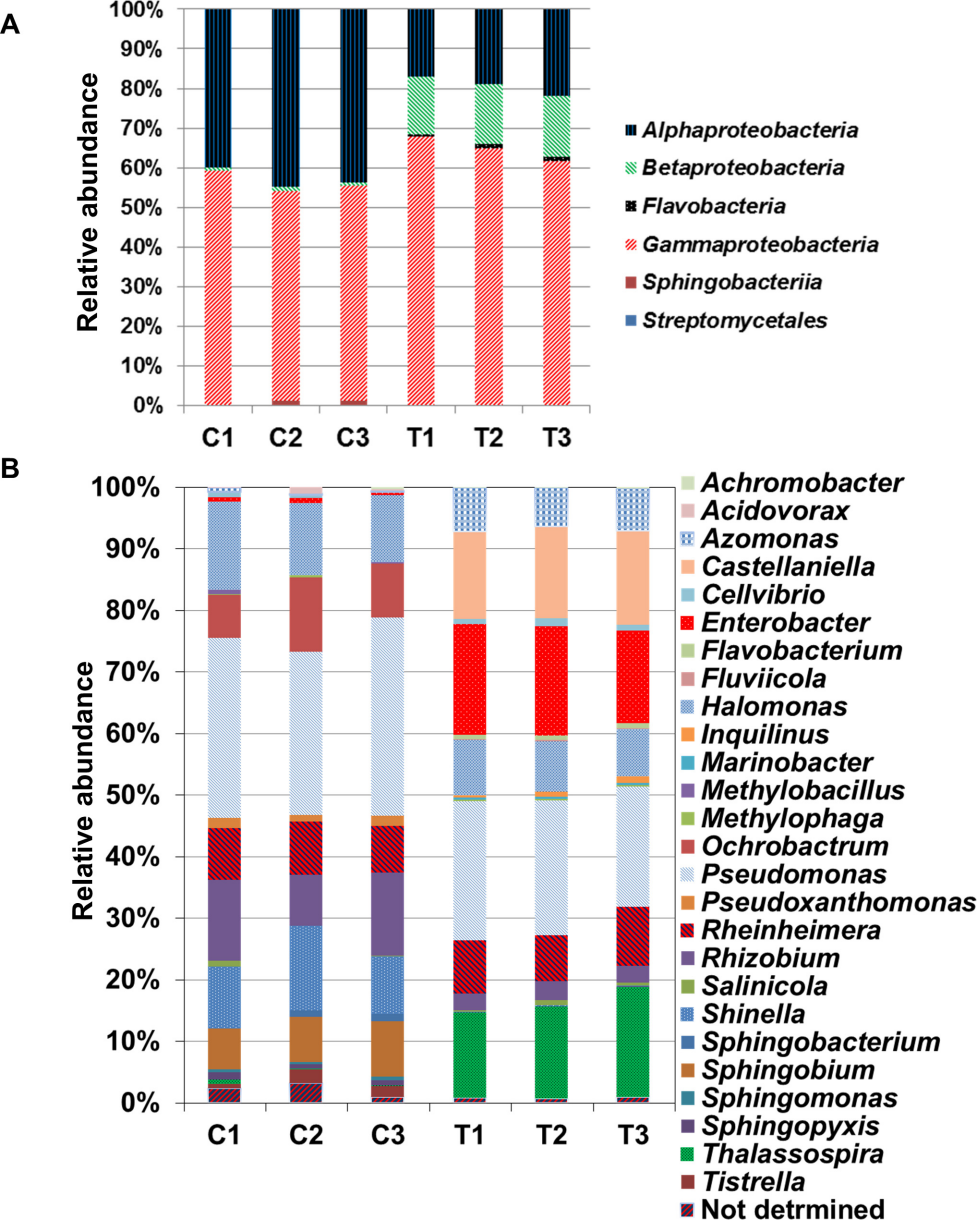
Relative abundance of different class (A) and genus (B) in each replica of the control (C1-3) and NaCl treated (T1-3) samples. The abundance is expressed as the percentage in the total number of reads per each OTU.

The proportional enrichment of *Alphaproteobacteria* class was high within the microbial community isolated from roots grown under normal conditions however, *Gammaproteobacteria*, *Betaproteobacteria* were dominant in response to salinity. In addition, salinity treatment leads to the appearance of *Flavobacteria* and *Streptomycetales* classes and disappearance of the *Sphingobacteria* class within the endophytic community (Fig 2A).

The analysis also revealed the presence of a total of 41 OTUs, representing 27 unique genera, where *Enterobacter*, *Halomonas*, *Marinobacter*, *Pseudomonas*, *Pseudoxanthomonas*, *Rhizobium* and *Thalassospira* spp. were represented more than once in the communities (Table 2, and Fig 2B). It is quite normal to find that the majority of the identified bacteria were assigned to *Proteobacteria* because it is the second largest known bacterial phylum and one of the major ones in soil [55]. This phylum included eight different *Pseudomonas* species (Table 2), some of which previously showed plant growth-promoting activity, including *P*. *aeruginosa* [56] and *P*. *stutzeri* [57]. It is noteworthy that many bacteria have 5 to 10 copies of 16S rRNA therefore, there is the possibility of sequence divergence between different copies of the gene from the same organism [58].

**Fig 3:**
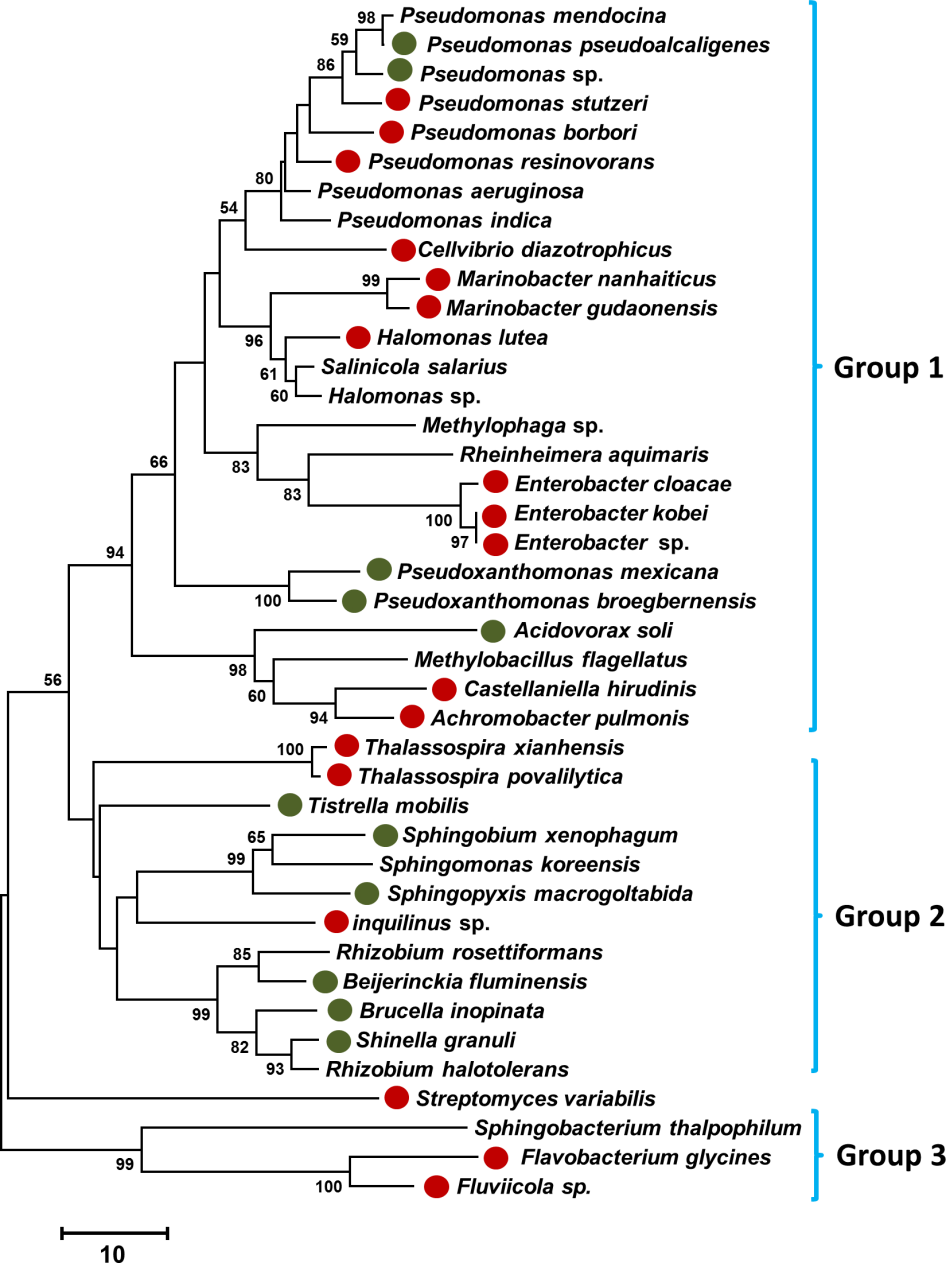
A neighbor-joining phylogenetic tree that was constructed based on the 16S rRNA DNA sequences, showing the relationships between the bacterial taxa identified in this study. The bootstrap values >50% (based on 1,000 replications) are shown at branching points. Differential abundance OTUs (*p* ≤ 0.05) in the salinity-treated and control roots are indicated by closed red and green circles, respectively.

The relationship between the *16S rRNA* gene sequences based on phylogenetic analysis showed that these sequences were clustered into three major groups, although the *Streptomyces variabilis* sequence was not among any of these clades because the unique 16S rRNA sequence belonged to the *Actinobacteria* phylum (Fig 3).

### The influence of salinity stress on the microbial community structure

The sequencing of the 16S rRNA library revealed the presence of a relatively low-divergence endophytic community, indicated as the number of identified OTUs (Table 3). The biodiversity and the richness of the bacterial endophytic communities were assessed based on the OTUs, Shannon, Simpson, Chao 1 and Goods Coverage indices. Because it was not recommended to use high impact treatment of data, especially when the coverage of sample diversity is not high[59], the raw abundance value was used without rarefying.

**Table 3:** Changes in the community richness and biodiversity indices among the six 16S rRNA libraries in response to NaCl treatment in Caliph medic. Significant changes based on *p* ≤ 0.05, n = 3, which were calculated using the one-way analysis of variance (ANOVA) test, are indicated by an asterisk.

Index	Control	NaCl-Treated	*p*-value
OTUs	29.33	34.33	0.03^*^
Chao1	31.25	34.83	0.21
Shannon	0.30	3.15	0.00^*^
Simpsoin	0.06	0.79	0.00^*^
Goods Coverage	99.99	99.99	0.56

The average number of OTUs and the evenness of species within the bacterial community were relatively low, but was significantly increased based on *p* ≤ 0.05, as indicated by the Shannon index, when plants were grown under saline conditions (Table 3). In addition, the probability that two randomly selected individuals in the habitat will belong to the same species was also increased, as indicated by the Simpson index under the same conditions; however, the estimated average richness for an OTU was unchanged in response to salinity stress, as indicated by the Chao 1 index (Table 3). The Goods Coverage index indicated that the samples were very well represented in the larger environment with an expectation value of more than 99% and this situation did not change in response to salinity (Table 3).

The low average number of the OTUs obtained from these communities is because of the fact that only a small number of the soil bacteria are facultative endophytes. Furthermore, the structure of a microbial community is altered based on the genetic basis of the microbial-host specificity, microbial-microbial interaction [60] and even microbial-tissue specificity (within the same host) [61], as well as based on the environmental conditions. For example, Dong and his colleagues [62] found that there are species-specific and inoculum-level variations in the ability of some bacteria to colonize *M*. *sativa* and *M*. *truncatula* seedlings.

The increase in the number of OTUs observed in the community, which were identified in salinity-stressed roots, is due to the alterations in the endophytic microbial ecosystem, which may also involve the presence of some opportunistic phytopathogens that may attack plants during periods when the plant is stressed. For example, *P*. *aeruginosa* is an opportunistic pathogen and was enriched in the communities isolated from the salinity treated roots [63]. The presence of some opportunistic phytopathogens may also associated with the appearance of some biocontrol microbes such as *P*. *stutzeri* (Table 2), a bacterium which has the ability to secrete hydrolytic enzymes against the *Fusarium solani* mycelia, the causative agent for the root rot disease in various plant species [64].

In fact, as a consequence of the salinity treatment, eight additional OTUs were observed within the community (*p* ≤ 0.05) while five other bacterial species disappeared (Table 2). For example, *Thalassospira xianhensis* and *Castellaniella hirudinis* were not represented in the bacterial communities isolated from the untreated roots while *Brucella inopinata* and *Beijerinckia fluminensis* were not represented in the communities of the salinity-treated roots (Table 2). Localization of the differentially enriched species of significant abundance (*p* ≤ 0.05) on the phylogenetic tree revealed that the three major clades of this tree equally embraced these bacteria, regardless of their species (Fig 3).

**Fig 4:**
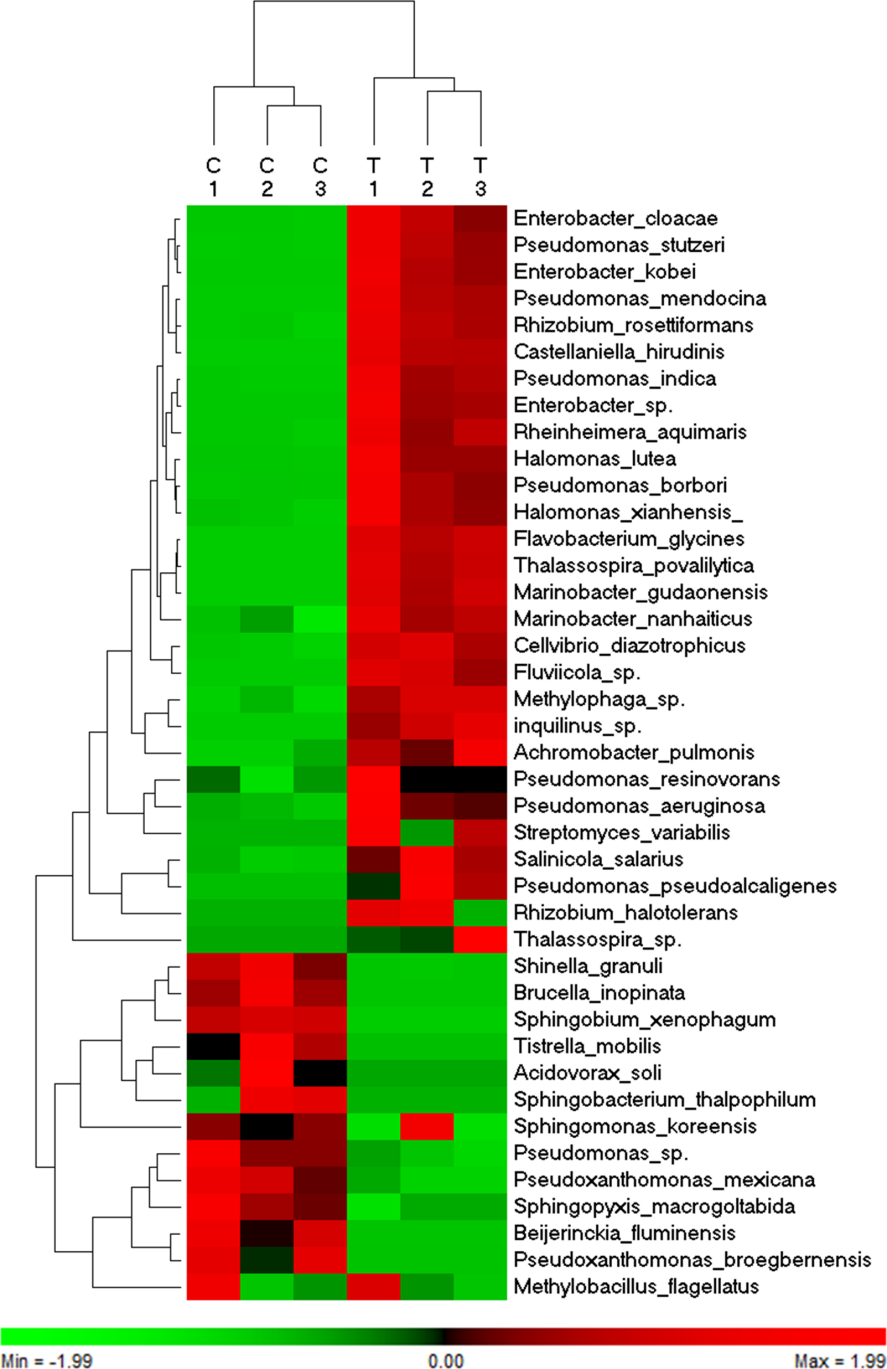
A heat map of the hierarchical cluster analysis and a dendrogram showing the normalized relative abundance of 41 identified species from three bacterial communities, which were prepared from roots grown under normal conditions (C1-3) and from three bacterial communities prepared from roots grown under NaCl stress (T1-3).

Hierarchical cluster analysis, based on the abundance of 41 bacterial species identified from six communities of roots grown in normal and saline conditions revealed that 39 species were clustered into two major groups (Fig 4). These groups shared a conserved abundance profile among the three libraries of the same treatment (control vs. salt). The first group included 28 OTUs with a high level of abundance in the bacterial communities identified in plants grown under saline conditions, whereas the second group included 11 OTUs with a high level of abundance in the communities identified in plants grown under normal conditions. Since they did not have a consistent richness among the three biological replicates, *Methylobacillus flagellates* was clustered out of the first group and the *Sphingomonas koreensis* was clustered out of the second group.

Differential enrichment analysis using the Mann-Whitney U test and based on the *p* ≤ 0.05 showed that, out of 41 OTUs identified in the bacterial community living in the roots, 29 were differentially enriched when the plants were exposed to salinity stress (Table 2), of which 10 were negatively affected and 19 were enriched due to salinity treatment. For example, *Brucella inopinata*, *Sphingobium xenophagum* and *Shinella granuli* were abundant in the root when grown under normal conditions. On the other hand, *Enterobacter kobei*, *Halomonas lutea*, *Thalassospira povalilytica* and *Pseudomonas stutzeri* were abundant in the root when grown under saline conditions.

The differentially enriched species in response to salinity included OTUs that were previously isolated from *M*. *sativa*, such as *Endobacter medicaginis* [26], and other OTUs were isolated from corn, such as *Enterobacter kobei* [65], and *Enterobacter cloacae* from date palm [6]. Previous studies showed that some strains of *Enterobacter* sp. are able to help plants growing under saline conditions by providing ACC deaminase, the phytohormone IAA and siderophores that facilitate iron acquisition under iron-limiting conditions.

It is noteworthy that other differentially enriched species identified in this study were previously isolated from marine and salt-polluted environments. For example, *Marinobacter gudaonensis* was isolated from oil-polluted saline soil [66] and a *Marinobacter nanhaiticus* strain was isolated from the sediment of the South China Sea [67]. The latter strain was able produce a suite of acylpeptidic marinobactin siderophores [68]. In addition, *Streptomyces variabilis* was isolated from the marine sponge *Iotrochota* sp. [69] and the *Halomonas lutea* sp. nov., which is a moderately halophilic bacterium, was isolated from a salt lake [70].

Some of the identified OTUs were assigned to nitrogen-fixing bacteria. For example, *Cellvibrio diazotrophicus* were isolated from the rhizosphere of salt meadow plants [71], *Beijerinckia fluminensis* from the giant reed and switchgrass rhizosphere [72] and acidic soil [73], and *P*. *stutzeri* [74] from chemically-stressed soil [75].

Identification of bacterial species from the *M*. *truncatula* roots, similar to those species isolated from saline and marine environments, is not surprising since the soil used in this experiment was mainly composed of a sandy texture (Table 1) and obtained from a field located near to the seashore; therefore, the microbial community within this soil would likely have been affected by the marine ecosystem.

The bacterial species identified in this study also included OTUs that potentially are useful in the environment. For example: *Thalassospira xianhensis* is a polycyclic aromatic hydrocarbon-degrading marine bacteria [76]; *Achromobacter pulmonis* was isolated from *Phragmites australis* (common reeds) and is able to remove carbamazepine [77]; *Pseudomonas resinovorans* is a carbazole- (CAR-) degrading bacterium [78]; *Thalassospira povalilytica* is a marine polyvinyl-alcohol degrading bacterium [79]; and *Streptomyces variabilis* is also a marine-derived bacteria, which produces the anti-cancer agent ammosamide [80].

**Fig 5:**
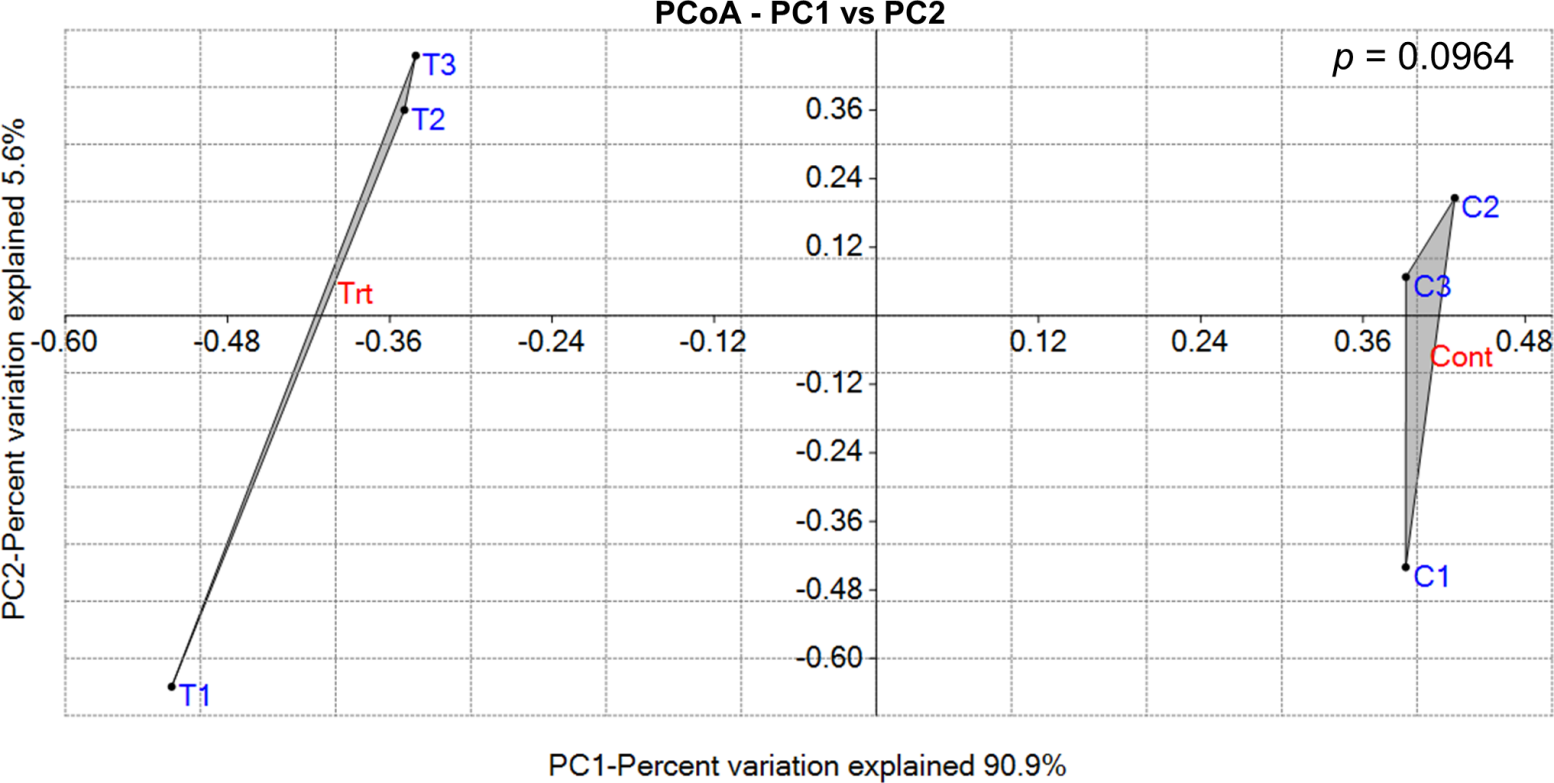
Principal Coordinate Analysis (PCoA) illustrating distances between bacterial communities identified from control (C1-C3) and NaCl-treated roots (T1-T3) of Caliph medic. The pairwise comparison using the ANOSIM test did not show significant variation (*p* = 0.0964) between the community groups identified from control plants (Cont) and NaCl plants (Trt). The first two coordinates explained about 96% while the third coordinate explained only 2.3% of the variation.

Despite the appearance of several new OTUs in response to salinity treatment, the pairwise overall variation analysis using the ordination-based and the ANOSIM similarity test approaches did not confirm the appearance of a significantly totally different endophytic community in Caliph medic roots in response to salinity stress (Fig 5).

Unlike free living soil microbes, salinity may have a minor effect on the endophytic communities since plants may buffer, to some extent, the deleterious effects of salinity on the roots’ ecosystem therefore, the low bacterial variation between the communities is not unexpected.

In order to confirm the presence of endophytic plant growth promoting bacteria in the Caliph medic roots of plants grown under normal and salinity conditions, these bacteria were isolated, identified and their ability to produce ACC-deaminase, ammonia and IAA were measured. Moreover, the ability of these isolates to solubilize zinc and phosphorus were tested. The results showed that the newly isolated bacteria belonged to the *Enterobacteriaceae* and *Pseudomonadaceae* family. The isolated bacteria from plants grown under normal condition included strains similar to *Enterobacter* and *Pseudomonas* species (S2A Table), while those strains isolated from the plants grown under saline condition included strains similar to *Enterobacter*, *Klebsiella* and *Pantoea* species (S2B Table). Several of the isolated strains analyzed in this study showed the ability to catalyze the hydrolysis of the ACC to ɑ-ketobutyrate, to produce ammonia and IAA or similar compounds, and to solubilize zinc and phosphorus, regardless their original source (from salinity treated or untreated roots) (S3 Table). Therefore, the bacterial community present in the internal parts of the roots of Caliph medic have the potential role to promote plant growth under both normal and saline conditions. Several isolates identified in this experiment were also identified using the cultivation independent approach, however, both approaches (cultivation dependent and independent) are incomparable since other factors such as the use of different culture media will affect the enrichment. Furthermore, the cultivation dependent method is not quantitative.

Despite the identification of this set of bacterial species from *M*. *truncatula* roots, other bacteria species may remain unidentified. This is because a bacteria-host symbiotic relationship depends on the nature of the soil and other environmental factors, such as temperature. Additionally, by using a common DNA extraction method, it is difficult to ensure the extraction of genomic DNA and, in turn, the barcoding of every endophytic bacteria species from the root, since some species can form refractory-coated spore-like structures that prevent complete DNA extraction [81].

In conclusion, we were able in this report to identify a bacterial community containing a wide range of known and unknown endophytic species that are affected by the saline conditions in root tissues. The information obtained from this project is important for the isolation and further molecular characterization of endophytes from the model plant *M*. *truncatula*.

## Supporting Information

S1 Table Oligonucleotides used in the *16S rRNA* gene amplification and barcoding. (DOCX)

S2 Table Endophytic bacterial strains isolated from Caliph medic roots when plant grew under normal (A) and saline conditions (B).
(XLSX)

S3 Table The capacity to solubilize minerals and the ability to produce ACC-deaminase, IAA and similar compounds by the newly isolated strains. Activity or product not detected in the assays is denoted by N.D.
(XLSX)
