# Supplementary material for: Impact of Soil Salinity on the Structure of the Bacterial Endophytic Community Identified from the Roots of Caliph Medic (Medicago truncatula)
Source: PLoS One. 2016 Jul 8;11(7):e0159007. doi: 10.1371/journal.pone.0159007 (PMC4938511; doi:10.1371/journal.pone.0159007)
Supplement: S1 Table — (DOCX) [file pone.0159007.s001.docx]

**S1 Table.** Oligo nucleotides used in the PCR for 16S rRNA gene amplification and barcoding.

| **Target** | **Forward multiplex identifier (MID) (5’-3’)** | **Forward Primer (5’-3’)** | **Reverse multiplex identifier (MID) (5’-3’)** | **Reverse Primer (5’-3’)** |
| --- | --- | --- | --- | --- |
| 16S, V1-V4 | AGCACTGTAG | GAGTTTGATCMTGGCTCAG | AGCACTGTAG | TACCAGGGTATCTAATCC |
|  | ATCAGACACG |  | ATCAGACACG |  |
|  | ATATCGCGAG |  | ATATCGCGAG |  |
